# Compression of the Thorax for Relieving the Pain of Pleurisy

**Published:** 1890-02-08

**Authors:** 


### THE PRACTITIONER'S OUTLOOK AND RETROSPECT.

[*The Editor will be glad to receive offers of co-operation and contributions from members of the profession. There is no doubt that much valuable material full of interest and instruction is at present lost to science. This is largely so in the case of* (1) *the younger and less-known members of the hospital staff* (2) *those connected with cottage hospitals, and* (3) *the great body of medical practitioners not attached to any institution. The co-operation of all is cordially invited to enable the Editor to make* THE HOSPITAL *of the utmost possible use and value to the profession. Specialists willing to review books on their special subjects are invited to communicate this fact at once. All letters should be addressed to* THE EDITOR, THE LODGE, PORCHESTER SQUARE, LONDON, W.]

### MEDICINE.

### COMPRESSION OF THE THORAX FOR RELIEVING THE PAIN OF PLEURISY.

Dr. Otto, of Berlin, warmly advocates the compression of the thorax, employed by Drs. D. Powell, F. Roberts, and others in this country, though he effects it in a somewhat different manner. Using a roller 6-7 cms. (2⅝ in.) wide, he carries it round the chest at the level of the seat of pain, in several turns overlapping one another from below upwards. Beginning at the axillary line of the sound side, he fastens the ends firmly one to another at this point, securing the turns of the roller by transfixing it at that opposite with a large safety pin, the patient having expanded his lungs to the utmost.

